# Preloop trial: study protocol for a randomized controlled trial

**DOI:** 10.1186/s13063-018-2977-9

**Published:** 2018-11-09

**Authors:** Elisa Mäkäräinen-Uhlbäck, Heikki Wiik, Jyrki Kössi, Pasi Ohtonen, Tero Rautio

**Affiliations:** 1 Oulu University Hospital, PL 10, 90029 OYS, Oulu, Finland; 2 Päijät-Häme Central Hospital, Keskussairaalankatu 7, 15850 Lahti, Finland

**Keywords:** Loop-ileostomy closure, Rectal cancer, Hernia prevention, Synthetic mesh, Biological implant

## Abstract

**Background:**

A temporary loop ileostomy, which is used to decrease the risk of symptomatic anastamotic leakage after anterior resection and total mesorectal excision (TME), is traditionally closed without any mesh. However, as 44% of incisional site hernias need further repair after stoma closure, attention has increasingly been paid to the use of mesh. Research on the prevention of these hernias is scarce, and no studies comparing different meshes exist.

**Method/Design:**

The Preloop trial (Clinical Trials NCT03445936) is a prospective, randomized, controlled, multicenter trial to compare synthetic mesh (Parietene Macro™, Medtronic, Minneapolis, MN, USA) and biological implants (Permacol™, Medtronic) at a retromuscular sublay position for the prevention of incisional site hernias after loop-ileostomy closure. The main endpoints in this trial are infections at 30-day follow-up and the incidence of hernias clinically or on CT scan at 10 months after closure of the stoma. The secondary endpoints are other complications within 30 days of surgery graded with the Clavien-Dindo classification, reoperation rate, operating time, length of stay, quality of life measured with RAND-36, and incidence of hernia over a 5-year follow-up period. A total of 100 patients will be randomized in a 1:1 ratio.

**Discussion:**

This is a pilot trial that will be undertaken to provide some novel evidence on the safety profile and efficiency of both synthetic mesh and biological implants for the prevention of incisional hernias after closure by temporary loop ileostomy. The hypothesis is that synthetic mesh is economical but equally safe and at least as effective as biological implants in hernia prevention and in contaminated surgical sites.

**Trial Registration:**

ClinicalTrials.gov, NCT03445936. Registered on 7 February 2018.

## Background and rationale

Anastomotic leakage is a major cause of morbidity after sphincter-saving anterior resection and total mesorectal excision (TME), with a reported incidence in a large meta-analysis of 11% [1]. A temporary diverting stoma has been shown to significantly decrease the risk of symptomatic anastomotic leakage and the need for reoperation in meta-analyses [2–5]. Defunctioning loop ileostomy is superior to loop colostomy in terms of a lower surgical complication rate and fewer parastomal and incisional hernias and surgical site infections [6, 7].

In a recently published retrospective study by Juratli et al., the incidence of incisional hernias after loop-ileostomy closure detected by computed tomography (CT) scan 12–24 months later reached 21.5% [8]. Another retrospective cohort showed an incisional hernia incidence of 13.5% after loop-ileostomy closure after a median follow-up of 20 months [9]. Of these clinically detected incisional hernias, 44% needed further surgical repair upon meta-analysis [10]. The true rate of incisional hernias might be higher, though, as the incidence of hernias increases over time [11, 12].

So far, the fear of infectious complications has discouraged the use of synthetic mesh in contaminated surgical fields. Nevertheless, the infection rate of patients with synthetic prophylactic mesh applied in contaminated surgical fields is similar to that of controls without the mesh at the time a permanent stoma was created [13–16]. The use of synthetic meshes as prophylaxis has also been shown to be safe in other contaminated abdominal surgery sites [15, 17].

There are only a few previous studies on prophylactic mesh utilization at the time of stoma closure. Biological mesh has been demonstrated to be safe in terms of complications [18, 19] and effective in preventing hernias [20]. However, no randomized controlled trials (RCTs) have been published. Reinforcement of Closure of Stoma Site (ROCSS) Collaborative and the West Midlands Research Collaborative has recently published a protocol for an RCT comparing a standard suture closure to mesh reinforcement with biological intra-abdominal mesh after stoma closure [21]. Research on synthetic meshes in the same context is even rarer. An Australian study group found a significant reduction in the rate of incisional hernias following mesh placement without increasing the rate of infection complications, despite the use of prosthetic material in the contaminated surgical site [22]. In a register study, the application of synthetic mesh was found to be both safe and effective in preventing incisional site hernias after stoma closure [23]. There have been no trials comparing biological implants and synthetic mesh on incisional hernia prophylaxis.

Macroporous monofilament polypropylene mesh (Parietene Macro™, Medtronic) has previously been shown to be safe in contaminated surgical sites according to satisfactory preliminary results of the PREVENT trial in parastomal hernia prophylaxis [24]. Likewise, there have been no trials on the use of porcine dermal collagen implants to prevent hernias, although they have been widely studied for ventral hernia repair [25].

The ideal location for the prophylactic mesh is still unclear. In a recent meta-analysis that included all types of postoperative ventral hernias, the sublay location of mesh was associated with the lowest rate of infection and recurrence [26]. Based on the results of a meta-analysis on parastomal hernia prevention, the sublay position of a non-absorbable prophylactic mesh is preferred [16].

### Objectives

The objective of the Preloop trial is to compare synthetic mesh (Parietene Macro™, Medtronic) and biological implants (Permacol™, Medtronic) for the prevention of incisional site hernias after temporary loop-ileostomy closure (see Table 1). Our hypothesis is that synthetic mesh is equally safe and effective as biological implants in incisional site hernia prophylaxis, and that they both can significantly reduce the rate of incisional hernias detected in previous studies. Its lower price also makes synthetic mesh a more lucrative option. By demonstrating that synthetic mesh is equal to biological implants in terms of safety and efficiency, this study might encourage investigators to design upcoming trials that use synthetic materials in contaminated fields. As previous studies are scarce, the Preloop trial was planned as a pilot study to provide preliminary results on the safety and efficiency of both devices used in the study.

**Table 1:** Comparison of devices in Preloop trial

Comparison of devices	Parietene Macro	Permacol
Manufacturer	Medtronic	Medtronic
Mesh type	Synthetic	Biological implant
Material	Macroporous monofilament polypropylene mesh	porcine dermal collagen implant
Size	Trimmed to fit retromuscular space, original size before trimming 10 × 15 cm	length 10 cm, width 5 cm

### Trial design

**Fig. 1:**
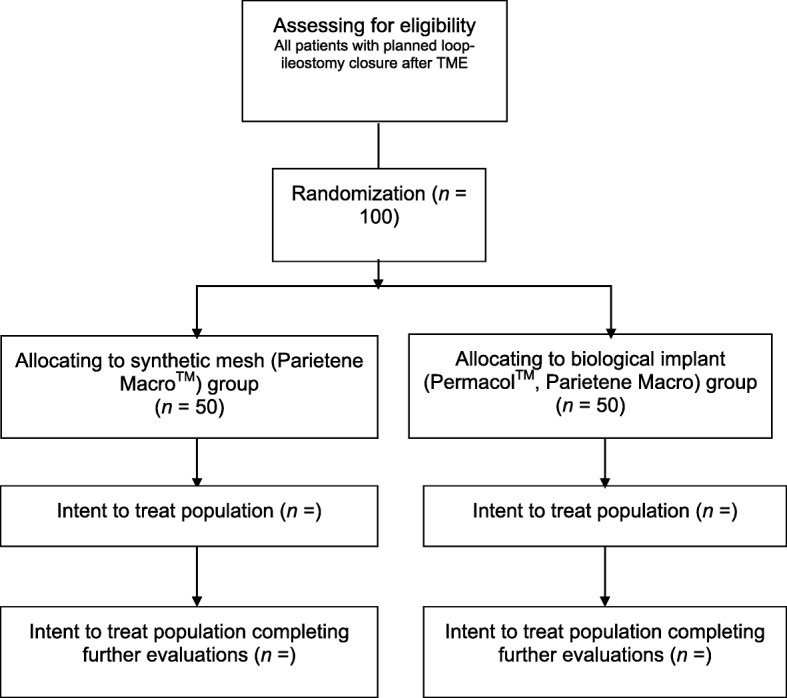
Flowchart

The Preloop trial (see also Fig. 1) is a prospective, randomized, controlled, multicenter study comparing two devices (synthetic mesh, Parietene Macro™, Medtronic; and biological implant, Permacol™, Medtronic) in preventing ileostomy site hernias, including in patients who have undergone low anterior resection and TME for rectal adenocarcinoma with diverting loop ileostomy. The trial is independent from any kind of industrial sponsorship.

### Devices trialed

Parietene Macro™ (Medtronic) is a macroporous monofilament polypropylene synthetic mesh widely used in hernia repair. Permacol™ is an acellular porcine dermal collagen implant designed for hernia repair (see Table 1). The size of the biological implant selected for this study (Permacol™, Medtronic) reflected a compromise of price. As the price of an implant increases significantly the larger it is, the chosen 10 cm in length and 5 cm in width represents a realistic option for use in general practice, *if* it is shown to be both effective and safe. The significantly more affordable synthetic mesh (Parietene Macro™, Medtronic) has been trimmed to fit the retromuscular space in width, as would be done in general everyday practice.

## Methods

### Study setting

This study will be completed in several university and central hospitals in Finland: Oulu University Hospital, Turku University Hospital, Tampere University Hospital, Kuopio University Hospital, Jyväskylä Central Hospital, and Seinäjoki Central Hospital.

### Eligibility criteria

#### Inclusion criteria

- Anterior resection and TME with temporary loop ileostomy for rectal adenocarcinoma without any adjuvant oncological treatment
- Age 18 years or older
- Life expectancy of at least 12 months
- Signed informed consent with agreement to attend all study visits
- Loop-ileostomy closure within 2–4 months after the primary operation

#### Exclusion criteria

- Presence of a comorbid illness or condition that would preclude surgical treatment (American Society of Anesthesiologists [ASA] grade 4–5).
- Concurrent or previous malignant tumors within 5 years before study enrollment
- Presence of T4b tumors necessitating a multi-organ resection
- Patients treated with postoperative adjuvant chemotherapy
- Patients undergoing emergency procedures
- Primary rectal surgery along with major concomitant procedures (e.g. hepatectomies, other intestinal resections).
- Metastatic disease with life expectancy of less than 12 months
- Pregnancy or suspected pregnancy

All subjects who have had anterior resection and TME for rectal adenocarcinoma at any of the study sites and have not received any adjuvant oncological treatment due to routine protocol of rectal cancer treatment will be considered for inclusion at 1-month follow-up at the outpatient department after the primary operation. The current practice at Oulu University Hospital is to close temporary loop ileostomy by suturing the fascial defect without a mesh at either 2–4 months after uncomplicated anterior resection or about 8–12 months after anterior resection after adjuvant oncological treatment has been accomplished. A full-body CT scan is part of the 1-year follow-up protocol after anterior resection for rectal adenocarcinoma. Therefore, only subjects not receiving adjuvant treatment can be enrolled in the study without having any additional radiation and still undergo a CT scan for both cancer follow-up and detection of possible incisional hernias about 1 year from each operation. After receiving proper information on the possible advantages and disadvantages of intervention, and after voluntarily signing the informed consent form, the subjects will be enrolled in the Preloop trial.

Participating investigators are qualified colorectal or general surgeons experienced in the surgical management of patients with temporary stomas. Further, all participating surgeons are experienced in the relevant technique for loop-ileostomy closure procedures and the use of retromuscular meshes. The technique that will be used to apply both the synthetic mesh and the biological implant is identical. Surgeon contributions to the study will be limited to no less than 10 cases per hospital, and all attending hospitals will be directed to assign the operations to specific surgeons to eliminate potential sources of bias.

### Interventions

#### Primary procedure

Loop ileostomy will be established at anterior resection on a site previously marked by a trained ostomy nurse. The bowel forming the stoma will be everted about 1–2 cm above the skin and attached by Monocryl 4–0 sutures to the edges of the skin.

#### Closure of stoma site

The closure of the temporary loop ileostomy will be performed 2–4 months after uncomplicated anterior resection on subjects who had since received no adjuvant treatment. The re-establishment of intestinal continuity will be achieved through the ostomy trephine either by staples or by hand-sewn anastomosis, according to the surgeon’s preference, and the bowel will be positioned back into the peritoneal cavity. Midline laparotomy will be used in the case of extensive bowel adhesions. The posterior rectus sheath will be closed with interrupted 2.0 polydioxanone sutures (PDS Plus Antibacterial Suture, Ethicon, Johnson & Johnson, Somerville, NJ, USA). The retromuscular space will be dissected with monopolar diathermy or another suitable energy device to create the space for the mesh. In the first group, a biological implant measuring 10 cm in length and 5 cm in width with the corners slightly curved (Permacol™, Medtronic) will be placed in the retromuscular space established as described above. In the second group, a lightweight polypropylene mesh (Parietene Macro™, Medtronic) with measures matching the retromuscular space will be placed similarly into the retromuscular sublay position. Four interrupted 2.0 polydioxanone sutures (PDS Plus Antibacterial Suture, Ethicon, Johnson & Johnson), one in each corner of the mesh, will be used to fix the mesh to the underlying posterior rectus sheath. After mesh fixation, the anterior rectus sheath will be closed with interrupted 0 polydioxanone sutures (PDS Plus Antibacterial Suture, Ethicon, Johnson & Johnson). The skin defect will be closed by a running subcutaneous purse-string suture with a 2.0 polyglactin thread (Coated Vicryl Plus Antibacterial Polyglactin 910 Suture, Ethicon, Johnson & Johnson).

As a preoperative antibiotic, prophylaxis cefuroxime 1.5 g and metronidazole 500 mg administered intravenously (i.v.) will be used. Patients with a body mass index (BMI) ≥ 30 will receive cefuroxime 3 g and metronidazole 1 g i.v. If the patient has had a previous allergic reaction to primary prophylactic antibiotics, clindamysin 600 mg i.v. (or, in case of BMI ≥ 30, clindamysin 900 mg i.v.) and ciprofloxacin 400 mg i.v. will be administered. No mechanical bowel preparation will be given.

#### Outcomes

The main endpoints of the study will be the surgical site infection rate and the incidence and extent of stoma site incisional hernia, either symptomatic or asymptomatic, detected clinically and/or radiologically 10 months after surgery. All patients will have had a full-body CT scan and will be met in the outpatient clinic, according to hospital protocol, 12–14 months after the anterior resection. During this visit, the condition of the previous loop ileostomy scar will be assessed, and a clinical estimation of the presence of incisional hernia will be made. All symptoms in this regard will be recorded. As a CT scan at about a 1-year follow-up is part of the follow-up protocol after anterior resection for rectal adenocarcinoma, patients will be exposed to no additional radiation within the trial, and therefore only subjects not receiving adjuvant therapy will be enrolled. All CT scans will be analyzed by the same radiologist, who will be blinded to the type of mesh used. The data collected will include exact measures of fascial defect, location of the defect, content and extent of the hernia sac, and the incidence of other hernias.

Surgical site infection has been defined in accordance with the Centers for Disease Control and Prevention (CDC) Surgical Site Infection Event. Any suspicion of surgical site infection or other complication during the primary hospital stay after loop-ileostomy closure will be assessed by an experienced colorectal/general surgeon who will not have been involved in the Preloop trial and will have been blinded to the mesh used to avoid bias. In the same manner, all concerns regarding complications or harmful events after hospitalization will be referred to second opinion.

The European Hernia Society definition of incisional site hernia has been applied [27], and the Clavien-Dindo classification of complications will be used to ensure proper classification. All data concerning operations during the hospital stay, recovery, and complications will be recorded on an electronical case report form (eCRF) designed for this study.

Costs will be monitored and analyzed in detail. Direct costs comprise the price of the mesh, the length of the hospital stay, and the treatment of possible complications. An estimation of the facilities used, including operating room time, will also be made. Indirect costs will mainly comprise the length of sick leave.

The secondary endpoints of the study are as follows:

- Clavien-Dindo grade I–V complications within 30 postoperative days
- Reoperation rate
- Operative time (minutes)
- Length of stay (LOS, days)
- Quality of life (QoL) measured by the 36-item RAND health survey (RAND-36)
- Incidence of hernia on long-term follow-up (5 years)

#### Pre-intervention data

- BMI
- ASA classification
- Other significant diseases and medications
- Smoking history
- Previous hernias, both symptomatic and asymptomatic, operated or unoperated
- Operative details on anterior resection, including anastomosis technique, distance from the anus, complications, and problems with the formation of stoma
- Tumor-node-metastasis (TNM) classification of the rectal cancer
- Quality of life measured by RAND-36 questionnaire

#### Intervention data

- Antibiotic prophylaxis used
- Operative time, operation room time, time to apply the mesh/implant
- Technique and approach used to close the stoma and re-establish intestinal continuity

#### Post-intervention data

- Length of hospital stay
- All complications measured by the Clavien-Dindo classification
- Surgical site infection classified by the CDC
- Demand for re-operation and mesh removal
- Re-admissions
- RAND-36 questionnaire
- Clinically and/or CT-detectable hernia
- Hemoglobin, carcinoembryonic antigen (CEA)
- Bowel function
- State of incisional wound for loop-ileostomy closure when leaving the hospital

#### Participant timeline

Randomization started in February 2018 at Oulu University Hospital and will last for 2 years. The follow-up schedule is presented in Table 2. Short-term results and complications will be monitored at discharge and 1 month after the operation. CT scans to detect subclinical hernias besides clinical evaluation will be performed 10 months after stoma closure as part of the routine protocol of rectal adenocarcinoma follow-up.

**Table 2:** Schedule of events

Schedule of events	Baseline	Procedure	Discharge	30 days ± 3 days	10 months ± 14 days	3 year ± 30 days	5 years± 30 days	Unscheduled visit
Informed consent	X							
Demographics and medical history	X							
QoL	X			X	X	X	X	
Procedure details		X						
CT scan findings					X			
Protocol deviation	X*	X*	X*	X*	X*	X*	X*	X*
Complications		X*	X*	X*	X*	X*	X*	X*
Study exit form							X**	

*Complete if applicable

**Complete when lost to follow-up, consent withdrawal, or when the subject has completed all study-related visits

*CT* computed tomography, *QoL* quality of life

#### Sample size

Two of the previously published retrospective studies reported the incidence of incisional hernias to be 13.5–21.5% on short-term follow-up after closure by suturing [8, 9]. Likewise, the number of hernias after closure with mesh has been very few in short-term follow-up [20, 22]. We assume that the hernia rate after closure by suturing detected in previous studies can be significantly diminished to about 5% with a prophylactic synthetic mesh or biological implant on long-term follow-up. The aim of the study is to generate preliminary results showing synthetic mesh is non-inferior to biological mesh. Assuming α = 0.05 and a power = 80% for incisional hernias and with 5% non-inferiority marginal, we would need 235 patients per group. Although previous studies on both biological implants and synthetic meshes have raised no concerns on the safety of the meshes [18–20, 22, 23], the use of synthetic meshes in contaminated surgical sites continues to be widely avoided in clinical practice, and no comparative studies on incisional hernia prophylaxis after temporary stoma closure exist. That said, we assume there is no difference in infectious complications between the meshes.

As this is a pilot study, each group will comprise 45 patients. Considering a possible 10% drop-out rate, our aim is to have 50 patients per group. Therefore, the estimated sample size of 50 in each group should give a reliable preliminary estimation of the safety and efficiency of both meshes for further studies.

#### Recruitment

All subjects fulfilling the inclusion criteria at each research site will be considered to participate in the trial at 1-month follow-up after anterior resection. An anonymous record will be kept prospectively on all subjects fulfilling the inclusion criteria but not attending, either by declining or for any other reason, for later assessment of selection bias.

### Assignment of interventions

#### Allocation

Subjects will be randomly allocated to study groups according to a computer-generated list compiled by a biostatistician otherwise uninvolved in the clinical care of trial patients.

Randomization will be performed in blocks, where the block size will vary randomly between two, four, and six. A separate randomization list will be created for each center, and randomization will be accomplished by electronic software. Randomization will be performed after confirmation of patient eligibility and willingness to participate.

#### Blinding

The subjects will be blinded to the method used during their hospital stay. After hospitalization, blinding will become impossible to maintain reliably due to a nationwide medical database directly accessible to patients. The surgeon who will meet the patients at 10-month follow-up will not be the same as the operating physician and will be blinded to the method used. The radiologist will also be blinded to the method used.

### Data collection, management, and analysis

#### Data collection methods

All data will be collected prospectively on an electronic database designed for this study. The RAND-36 questionnaire to be used in this study has been well documented previously.

The reasons for withdrawal will be documented carefully. The investigator will attempt to contact the subject at least three times prior to designating them as lost to follow-up. The investigator will document the date and type of attempted communication. If a subject cannot be reached during the visit window, a missed visit will be recorded; after three consecutive missed visits, the subject will be considered lost to follow-up, and a study exit form will be completed in the eCRFs. Any data on subject participation and procedures until their withdrawal will be analyzed within the research.

#### Data management

All data will be handled with utmost confidentiality. Permission for the study register has been submitted to Oulu University Hospital, and a description of the register has been provided.

#### Data monitoring

There is no data monitoring committee named in this trial. All complications and harmful events will be carefully reported using specific eCRFs, and serious events will be reported to the principal investigator immediately. As previous studies on synthetic mesh in particular are scarce, a preliminary safety analysis at 30-day follow-up will be made on 20 patients on each group. Any serious concern raised at any point in the study on the safety of the meshes will result in the consideration of preliminarily aborting the study.

#### Statistical methods

The main endpoints are the presence (proportion) of stoma site incisional hernias with 95% confidence intervals for both groups at 10-month follow-up, and the incidence of surgical site infection at 30 days after the closure of the stoma. Secondary outcomes are the presence of stoma site incisional hernias at 3- and 5-year follow-up, the relative improvement to QoL during the follow-up, Clavien-Dindo classification of complications at 30 days postoperatively, operation time, length of hospital stay, and reoperation rate.

Between-group comparisons of continuous variables will be performed by Student’s *t* test or Mann-Whitney *U* test, the latter of which will be used if heterogeneous variances persist. Categorical data will be compared using χ^2^ or Fisher’s exact test. Repeatedly measured continuous data will be analyzed by a linear mixed model (LMM) using individuals as random effects. The covariance pattern for the LMM was chosen according to Akaike’s information criteria. Two-tailed *p* values will be reported. Analyses will be performed using SPSS (version 24 or higher) (IBM Corp., Armonk, NY, USA) for Windows and SAS (version 9.4 or higher) (SAS Institute, Cary, NC, USA).

### Ethics and dissemination

#### Research ethics approval

This study follows the Declaration of Helsinki on medical protocols and ethics, and the study’s protocol has been approved by the Ethics Committee of Oulu University Hospital (reference number 2/2018). Each participating hospital applied for study permission at their unit.

#### Protocol amendments

Important protocol modifications will be communicated to the Oulu University Hospital Ethics Committee by amendments. All modifications will also be registered at Clinical Trials.

#### Confidentiality

Patient confidentiality will be strictly maintained. Patients will be pseudonymized by study identification numbers, and all data will be handled without using names or personal social security numbers. Access to patient records will be limited to the study group and the investigator-delegated study coordinator.

## Discussion

The true rate of incisional site hernias after temporary loop-ileostomy closure may be underestimated. The incidence ranges from 0 to 50% in previous studies, and up to 44% of these hernias might need further operation [10]. The rising trend of sphincter-saving procedures may increase the need to temporary stomas and their closure in the near future. Yet, limited data have been published on the appropriate technique for hernia prevention after temporary stoma closure. Previously, synthetic mesh was contraindicated in contaminated surgical fields, but there is growing evidence for the safe prophylactic use of synthetic meshes in contaminated surgical sites [17, 28]. Previous studies on hernia prevention after temporary stoma closure have compared the use of either synthetic or biological mesh to conventional suture closure, showing a significant reduction in hernia incidence. Additionally, hernia prevention with either synthetic mesh or biological implants is becoming increasingly popular and more utilized in gastrointestinal and abdominal wall surgery. Therefore, the current trial will focus on generating preliminary information on the most economic, effective, and safe method to prevent incisional hernias by comparing synthetic mesh to biological implants.

Blinding of the study is not possible In case of severe complications requiring re-operation, it is crucial to have direct access to all technical aspects of primary operation at all times. Patients will be blinded to the method used during their hospital stay, but it is impossible to control blinding after discharge due to the patient-accessible national medical database, which includes all medical records regarding patients.

This is the first randomized, controlled study to compare biological implants and synthetic mesh to prevent incisional hernias after loop-ileostomy closure. The selected group of patients will be as homogenous as possible, since they all will have undergone a low anterior resection for rectal adenocarcinoma before enrollment. We assume there will be no unexpected complications due to mesh reinforcement, and that a significant number of hernias can be prevented with either synthetic mesh or biological implants. As previous studies are still few, the Preloop trial was designed as a pilot trial to provide preliminary information to form the basis for future trials.

Biological implants have previously been widely considered the first choice for contaminated surgical fields despite their high price. If synthetic mesh can be shown to be equally safe and effective in preventing hernias, it will also become a more lucrative choice not only for preventing incisional site hernias after temporary stoma closure, but also for future trials on hernia prevention in other contaminated surgical sites. This research will provide novel preliminary information on both the short- and long-term effects and safety of the two prophylactic devices studied to prevent incisional site hernias after stoma closure.

### Trial status

Approval by the Ethics Committee at Oulu University Hospital was received (reference 2/2018), and patient recruitment at Oulu University Hospital started in February 2018. Seinäjoki Central Hospital enrolled its first patients in March 2018, and other hospitals will start the trial during autumn 2018.

## Abbreviations

ASA: American Society of Anesthesiologists
BMI: Body mass index
CDC: Centers for Disease Control and Prevention
CEA: Carcinoembryonic antigen
CT: Computer tomography
eCRF: Electronic case report form
i.v.: Intravenous
LOS: Length of stay
PDS: Polydioxanone sutures
QoL: Quality of life
RAND-36: 36-item RAND health survey
RCT: Randomized controlled trial
ROCSS: Reinforcement of closure of stoma site
TME: Total mesorectal excision
TNM: Tumor-node-metastasis
